# Rosiglitazone Adjunct to Sertraline for Major Depressive Disorder: A Randomized, Double‐Blind, Placebo‐Controlled Trial

**DOI:** 10.1155/da/5812795

**Published:** 2026-07-17

**Authors:** Ahmad Shamabadi, Reihane Karami, Sherinaz Sadati, Kimia Farahmand, Mohamadjavad Ershadmanesh, Rozhin Moosavi, Fatemeh Rahiminejad, Mohammad-Reza Khodaei Ardakani, Shahin Akhondzadeh

**Affiliations:** ^1^ Psychiatric Research Center, Roozbeh Psychiatric Hospital, Tehran University of Medical Sciences, Tehran, Iran, tums.ac.ir; ^2^ Department of Psychiatry, University of Social Welfare and Rehabilitation Sciences, Tehran, Iran, uswr.ac.ir

**Keywords:** combination drug therapy, glucose metabolism, insulin sensitizers, peroxisome proliferator activated receptor gamma agonist, thiazolidinediones

## Abstract

Insufficient responses to available treatments for major depressive disorder (MDD) underscores the necessity for innovative therapeutic strategies. This study investigated the efficacy of adjunctive rosiglitazone, an antidiabetic agent with neuroprotective and anti‐inflammatory effects, for MDD. In this 6‐week randomized, parallel‐group, double‐blind, placebo‐controlled clinical trial, patients with MDD were assigned to receive sertraline along with either rosiglitazone 2 mg or a matched placebo q12 h. Participants were evaluated using the Hamilton depression rating scale (HDRS) at baseline and weeks 2, 4, and 6. The difference in its score changes to the endpoint was the primary outcome. Side effects were documented as well. The participants had comparable baseline characteristics. A significant time –treatment interaction effect was observed for HDRS scores ($\eta_{P}^{2}$ = 0.052), with the rosiglitazone group experiencing significantly greater reductions at weeks 2 (Cohen’s *d* = 0.507), 4 (Cohen’s *d* = 0.556), and 6 (Cohen’s *d* = 0.541) compared to the placebo group. Additionally, the rosiglitazone group demonstrated significantly higher response rates until weeks 4 (50.0% vs. 24.2%) and 6 (84.4% versus 51.5%). By week 6, the remission rate was nonsignificantly higher in the rosiglitazone group (18.8%) than in the placebo group (9.1%). Side effect frequencies were comparable. In conclusion, rosiglitazone was beneficial for depressive symptoms of patients with MDD safely and tolerably. Further long‐term, large‐scale studies are recommended to confirm this evidence.

**Trial Registration:** ClinicalTrials.gov identifier: IRCT20090117001556N152

## 1. Introduction

Pharmacotherapy serves as an essential component in the first‐line treatment of moderate to severe major depressive disorder (MDD); however, current medications have some limitations. The response rate varies among participants, and about one‐third of patients with depression are treatment‐resistant [1]. Also, most Food and Drug Administration‐approved antidepressants take a few weeks to show effects [2]. Furthermore, these medications have some side effects, which can lead to poor adherence to treatment [3]. Electroconvulsive therapy is an option for treatment‐resistant cases and individuals needing urgent intervention, though it carries complications such as cognitive impairment [4] and a response rate of about 41% in those patients [5]. These challenges highlight the need for more effective and long‐lasting novel treatments, prompting researchers to explore new strategies like adjunctive therapy and drug repositioning.

Rosiglitazone is an agonist of peroxisome proliferator‐activated receptor (PPAR)‐γ, a nuclear transcription factor that regulates gene expression [6]. It has insulin‐sensitizing and anti‐inflammatory properties, reducing cytokines like tumor necrosis factor (TNF)‐α and interleukin (IL)−6 [7]. Studies suggest that inflammation and the PPAR‐γ‐ adiponectin axis in the adipose tissue contribute to depression pathophysiology [6, 8]. Furthermore, rosiglitazone is neuroprotective, decreasing neuronal apoptosis and inducing neurogenesis [9, 10]. These features make rosiglitazone a potential candidate for depression treatment. A pilot, open‐label, noncontrolled design study in 2010 on a small number of nondiabetic insulin‐resistant patients with depression found that adjunctive therapy with rosiglitazone significantly declined depression severity after 12 weeks. Interestingly, the depression severity reduction was not associated with improvements in metabolic markers caused by the treatment [11]. Animal studies have also examined the impact of rosiglitazone on stress‐induced depression and pain‐induced depression; the results indicated an antidepressant effect [10, 12]. Another PPAR‐γ agonist, pioglitazone, has also demonstrated significant antidepressant effects, both as monotherapy and in augmentative therapy, in various clinical studies among patients with or without metabolic disturbances [13–15]. Therefore, rosiglitazone might be beneficial in reducing symptom severity in individuals with MDD, regardless of their metabolic abnormalities. In this regard, the current randomized, double‐blind, placebo‐controlled clinical trial aimed to assess the effectiveness, safety, and tolerability of rosiglitazone as an adjunct to sertraline on moderate‐to‐severe MDD during a 6‐week treatment period.

## 2. Materials and Methods

The study protocol was published before the commencement with the Iranian Registry of Clinical Trials, a primary registry within the World Health Organization Registry Network, on June 20, 2023. The trial was designed, conducted, and reported in accordance with the Consolidated Standards of Reporting Trials framework (Appendix A) [16].

### 2.1. Design and Setting

This clinical trial employed a 6‐week, randomized, double‐blind, placebo‐controlled, parallel‐group design, and was conducted on patients with MDD between July 2023 and March 2025. The outpatient clinics of Roozbeh Hospital and Razi Hospital, two prominent psychiatric facilities affiliated with the Tehran University of Medical Sciences and the University of Social Welfare and Rehabilitation Sciences, served as the study sites.

### 2.2. Ethics

The study was conducted in accordance with the ethical standards set forth in the Declaration of Helsinki [17]. The protocol was approved by the School of Medicine Ethics Committee at the Tehran University of Medical Sciences on April 14, 2023 (identifier: IR.TUMS.MEDICINE.REC.1402.150). Participants provided written informed consent and were informed of their right to withdraw consent at any time without impacting standard medical support or their professional relationship with clinicians.

### 2.3. Participants

Eligible participants were those aged 18–60 years with a diagnosis of MDD confirmed by a senior psychiatrist using the Structured Clinical Interview for the fifth edition of the Diagnostic and Statistical Manual of Mental Disorders (DSM‐5) [18]. Participants were required to have a Hamilton depression rating scale (HDRS) [19] score of >22 for inclusion.

Patients with psychosis, suicidal ideation, other psychiatric disorders based on DSM‐5, cardiovascular or thyroid diseases, lactation, or pregnancy, the use of any psychoactive medication, a history of antidepressant receipt in the last month, a history of electroconvulsive therapy in the past 2 months, the need for drugs with serious interactions with sertraline and thiazolidinediones [20], and history of hypersensitivity to sertraline and thiazolidinediones were not included. No antidepressant regimen was changed to qualify for inclusion. Furthermore, any severe side effects or concurrent use of other antidepressants, mood stabilizers, antihistamines, antipsychotics, or supplements in the duration of the trial led to exclusion.

### 2.4. Interventions

Initially, all participants received 50 mg of sertraline daily, with the dosage being increased by 25 mg at weekly intervals, up to a maximum of 100 mg daily. This standardized sertraline regimen was intended to minimize the impact of different medications on therapeutic outcomes and side effects. Participants were also randomly assigned to take either 2 mg of rosiglitazone or a matched placebo q12 h over 6 weeks.

To improve medication adherence, dosette boxes were provided to participants at each session. Compliance was tracked through participant and family member reports as well as by counting the returned tablets. Noncompliance was defined as returning >30% of the tablets or reporting instances of missed doses. Participants who demonstrated noncompliance did not receive additional medication but remained in the study for all other aspects.

Using other interventions and alcohol were strictly limited.

### 2.5. Sample Size

A total of 40 participants per group was considered adequate to identify a between‐group difference of 3.5 points in the HDRS score reduction, assuming a standard deviation of 3.5. This calculation was based on a two‐tailed *t*‐ test for mean differences, with 90% statistical power, a 5% significance threshold, and an anticipated dropout rate of 20%, while also maintaining the generalizability of the results.

### 2.6. Randomization, Allocation Concealment, and Blinding

Randomization, allocation concealment, and blinding were managed by independent personnel. Participants were assigned to groups in a 1:1 ratio using a permuted block randomization method with block sizes of four. No stratification was applied during the randomization process. To maintain allocation concealment, assignment codes were kept in sequentially numbered, sealed, and opaque envelopes. Furthermore, the rosiglitazone and placebo tablets were identical in appearance, texture, and sensory characteristics, ensuring that participants, healthcare providers, HDRS raters, and outcome assessors remained blinded throughout the study.

### 2.7. Tools and Assessments

Baseline data on gender, age, marital status, literacy level, smoking status, and disorder duration were collected at the initial evaluation.

Depressive symptoms were assessed with the HDRS at baseline and at weeks 2, 4, and 6. This scale, established by Hamilton in 1960, is a gold standard for evaluating depression symptoms in MDD [19] and has been widely applied in research involving Iranian patients [21–23]. Before the study, the raters, who were two psychiatrists, met with the principal investigator to discuss the HDRS in detail, to review a set of educational clinical slides on the HDRS, and to further familiarize themselves with the HDRS in order to ensure high inter‐rater reliability in terms of patient ratings. There was an inter‐rater reliability of more than 90% between the two raters in the two centers in scoring using this scale.

Side effects were recorded through both a checklist and open‐ended questions to ensure comprehensive reporting by patients and their families [21–23]. A dedicated hotline was also provided for the real‐time reporting of any complications.

### 2.8. Outcomes

The main outcome measured was the difference between the groups in the change of HDRS scores from baseline to the study endpoint. Comparing early improvement (defined as ≥20% reduction in HDRS scores after 2 weeks [24]), response rates (defined as ≥50% reduction in HDRS scores) [25] at follow‐up sessions, the time needed to respond, remission rates (a total score of ≤7 in the HDRS [25]) at follow‐up sessions, HDRS score changes from baseline to follow‐up sessions, and the frequency of side effects between the groups were the secondary outcomes.

### 2.9. Statistical Analyses

Statistical analyses were conducted using IBM SPSS Statistics version 27 (IBM, Armonk, NY, USA). A significance threshold of 0.05 was applied. The Shapiro –Wilk test and Q –Q plots confirmed that the distributions of continuous variables adhered to normality. Analyses included patients who completed the trial or withdrew after week 2, with the exception of safety analyses, which included all participants who received at least one dose of rosiglitazone or placebo.

Categorical variables were presented as frequencies (*n*) and percentages (%) and compared using the chi‐square test. The response time between the groups was analyzed using Kaplan –Meier survival estimates with the log‐rank test [22, 23]. Censoring was applied for participants who withdrew or ended the study prior to experiencing the event of interest. Continuous variables were expressed as the mean ± SD and compared using the two‐tailed, independent‐samples *t*‐ test . Effect sizes for these *t*‐ tests were calculated using Cohen’s *d*, where values of 0.2, 0.5, and 0.8 correspond to small, moderate, and large effects, respectively [26]. The general linear model repeated‐measures analysis of variance assessed the effects of time and time‐treatment interactions on HDRS scores. When the assumption of sphericity was not met (ε < 0.75), Greenhouse‐Geisser adjustments were applied to correct the results of within‐subjects effects tests. Partial *eta* squared ($\eta_{P}^{2}$) was used to calculate effect sizes for one‐way repeated‐measures analysis of variance, where values of 0.0099, 0.0588, and 0.1379 corresponded to small, moderate, and large effects, respectively [26].

## 3. Results

**Figure 1:**
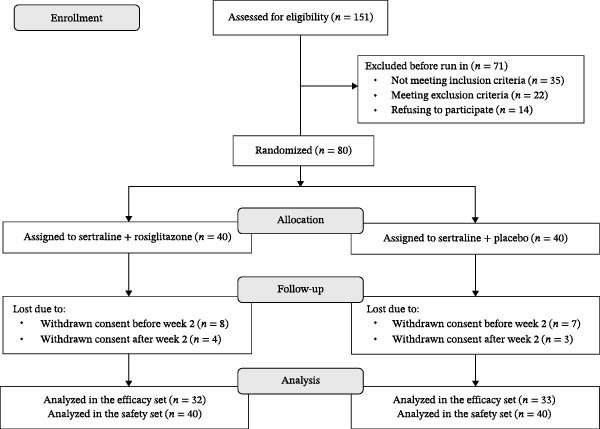
Flow diagram illustrating the process of patient selection for the trial and subsequent data analysis. All efficacy analyses were conducted on all randomized participants who participated in at least one postbaseline visit (32 in the sertraline + rosiglitazone group and 33 in the sertraline + placebo group). Analyses of safety data were conducted on the safety population, defined as all randomized participants who received at least one dose of their medication (40 in each group).

The diagram in Figure 1 depicts the stages of participant selection and highlights the reasons for any dropouts. The baseline characteristics of the participants included in the efficacy analyses are summarized and compared in Table 1.

**Table 1:** The baseline characteristics of the patients.

Variable		Sertraline + rosiglitazone (*n* = 32)	Sertraline + placebo (*n* = 33)	*p* ^d^
Gender, *n* (%)	Male	16 (50.0%)	16 (48.5%)	0.903^a^
	Female	16 (50.0%)	17 (51.5%)	
Age, mean years ± SD		33.56 ± 7.36	35.81 ± 7.31	0.220^b^
Marital status, *n* (%)	Single	8 (25.0%)	8 (24.2%)	0.855^c^
	Married	12 (37.5%)	13 (39.4%)	
	Separated	5 (15.6%)	3 (9.1%)	
	Widow	7 (21.9%)	9 (27.3%)	
Literacy level, *n* (%)	Primary school	7 (21.9%)	5 (15.2%)	0.890^a^
	Secondary school	7 (21.9%)	9 (27.3%)	
	Diploma	10 (31.3%)	10 (30.3%)	
	Higher education	8 (25.0%)	9 (27.3%)	
Disorder duration, mean years ± SD		4.64 ± 2.83	4.56 ± 3.04	0.913^b^
Smoker, *n* (%)		12 (37.5%)	10 (30.3%)	0.540^a^

Abbreviations: *n*, number; SD, standard deviation.

^a^Pearson chi‐square.

^b^Two‐tailed independent‐samples *t*‐test, equal variances assumed.

^c^Fisher’s exact test.

^d^No *p* was significant.

### 3.1. Depressive Symptoms

Table 2 gives a detailed breakdown of HDRS scores at baseline and each subsequent follow‐up session and changes in scores from baseline to each session.

**Table 2:** The comparison of Hamilton depression rating scale scores between the groups.

Time	Sertraline + rosiglitazone (*n* = 32), mean ± SD	Sertraline + placebo (*n* = 33), mean ± SD	*p*	Cohen’s *d*
At baseline	26.00 ± 3.10	26.36 ± 2.92	0.628^a^	0.121
At week 2	15.59 ± 4.12	20.42 ± 3.33	0.053^a^	0.489
Changes from baseline to week 2	−7.41 ± 2.89	−5.94 ± 2.89	0.045^ab^	0.507
At week 4	14.00 ± 3.33	16.30 ± 3.85	0.012^ab^	0.639
Changes from baseline to week 4	−12.00 ± 3.44	−10.06 ± 3.54	0.029^ab^	0.556
At week 6	10.19 ± 3.43	12.76 ± 4.10	0.008^ab^	0.979
Changes from baseline to week 6	−15.81 ± 3.90	−13.61 ± 4.24	0.033^ab^	0.541

Abbreviations: *n*, number; SD, standard deviation.

^a^Two‐tailed independent‐samples *t*‐ test, equal variances assumed.

^b^Statistically significant.

**Figure 2:**
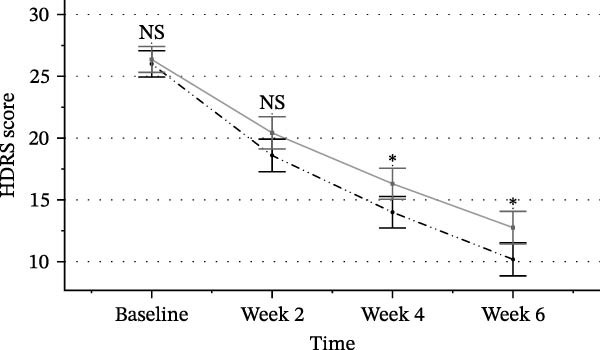
Repeated‐measures analysis comparing the effects of sertraline + rosiglitazone (black dotted lines with circles) versus sertraline + placebo (gray solid lines with squares) on mean Hamilton depression rating scale (HDRS) scores throughout the trial. Error bars indicate 95% confidence intervals. An asterisk ( ^∗^) denotes a statistically significant difference (*p* < 0.05) between the groups at each time point based on independent‐samples *t*‐ tests, while NS indicates nonsignificant differences.

A significant effect of time on HDRS scores was observed (*F* = 565.343, *df* = 2.027, *p*  < 0.001, $\eta_{P}^{2}$ = 0.900 Greenhouse‐Geisser corrected), denoting that the groups had improvements in depressive symptoms throughout the study. In addition, a significant time –treatment interaction effect was observed (*F* = 3.425, *df* = 2.027, *p* = 0.035, $\eta_{P}^{2}$ = 0.052 Greenhouse‐Geisser corrected), illustrating that the improvements varied between the groups (Figure 2), with the sertraline + rosiglitazone group showing significantly more reductions until all follow‐up sessions compared to the sertraline + placebo group (Table 2).

Table 3 outlines the number of patients who achieved a response in steps of 25% HDRS score reductions across follow‐up sessions for both groups. Additionally, the comparisons of early improvement, response, and remission rates are detailed in Table 4. As can be inferred from Tables 3 and 4, five patients in each group had a reduction of ≥20 and <25% in their scores by week 2. Furthermore, the sertraline + rosiglitazone group demonstrated a slightly faster mean ± standard error response time (4.74 ± 0.22 weeks) compared to the other group (5.06 ± 0.25 weeks; *p* = 0.381).

**Table 3:** The frequency of treatment responders in steps of 25% Hamilton depression rating scale score reductions between the groups until each follow‐up session.

Time	Group	<25% reduction, *n* (%)	25–49.9% reduction, *n* (%)	50–74.9% reduction, *n* (%)	≥75% reduction, *n* (%)
Week 2	Sertraline + rosiglitazone (*n* = 32)	14 (43.8%)	17 (53.1%)	1 (3.1%)	0
	Sertraline + placebo (*n* = 33)	21 (65.6%)	11 (34.4%)	0	0
Week 4	Sertraline + rosiglitazone (*n* = 32)	1 (3.1%)	15 (46.9%)	16 (50.0%)	0
	Sertraline + placebo (*n* = 33)	3 (9.1%)	22 (66.7%)	8 (24.2%)	0
Week 6	Sertraline + rosiglitazone (*n* = 32)	0	5 (15.6%)	25 (78.1%)	2 (6.3%)
	Sertraline + placebo (*n* = 33)	0	16 (48.5%)	15 (45.5%)	2 (6.1%)

Abbreviation: n, number.

**Table 4:** The comparison of early improvement, response to treatment, and remission rates between the groups.

Variable	Sertraline + rosiglitazone (*n* = 32)	Sertraline + placebo (*n* = 33)	*p*
Early improvers *n* (%)	23 (71.9%)	16 (48.5%)	0.077^a^
Responders at week 2, *n* (%)	1 (3.1%)	0	0.492^a^
Remission at week 2, *n* (%)	0	0	^b^
Responders at week 4, *n* (%)	16 (50.0%)	8 (24.2%)	0.041ac
Remission at week 4, *n* (%)	0	0	^b^
Responders at week 6, *n* (%)	27 (84.4%)	17 (51.5%)	0.007^ac^
Remission at week 6, *n* (%)	6 (18.8%)	3 (9.1%)	0.303^a^

Abbreviation: n, number.

^a^Fisher’s exact test.

^b^No statistics were computed as the variable was a constant.

^c^Statistically significant.

### 3.2. Safety and Tolerability

Throughout the study, no severe adverse events requiring participant exclusion occurred, and all participants adhered to their prescribed medication regimens. Additionally, the frequency of adverse events was similar between the groups, as detailed in Table 5. The most common side effects were diarrhea (15.0%), bloating (12.5%), and headache (12.5%) in the sertraline + rosiglitazone group and diarrhea (15.0%), bloating (12.5%), abdominal pain (12.5%), and headache (12.5%) in the sertraline + placebo group, while no significant differences were observed between the groups.

**Table 5:** The comparison of the frequency of adverse events between the groups.

Side effect	Sertraline + rosiglitazone (*n* = 40)	Sertraline + placebo (*n* = 40)	*p* ^ab^
Diarrhea, *n* (%)	6 (15.0%)	4 (10.0%)	0.737
Weight gain, *n* (%)	4 (10.0%)	3 (7.5%)	1.000
Bloating, *n* (%)	5 (12.5%)	4 (10.0%)	1.000
Abdominal pain, *n* (%)	4 (10.0%)	4 (10.0%)	1.000
Increased appetite, *n* (%)	4 (10.0%)	3 (7.5%)	1.000
Headache, *n* (%)	5 (12.5%)	4 (10.0%)	1.000
Nausea, *n* (%)	4 (10.0%)	3 (7.5%)	1.000
Vomiting, *n* (%)	3 (7.5%)	3 (7.5%)	1.000

Abbreviation: n, number.

^a^Fisher’s exact test.

^b^No *p* was significant.

## 4. Discussion

In this 6‐week clinical trial, adding rosiglitazone 2 mg twice daily to sertraline was superior to adjunctive placebo for symptom improvement and disorder severity among patients with moderate to severe MDD. The reductions in HDRS scores were significantly greater in the rosiglitazone group compared to those in the placebo group after 2, 4, and 6 weeks of follow‐up. The responses to treatment were also better at weeks 4 and 6 in the rosiglitazone group. Besides, rosiglitazone was found to be safe and well‐tolerated in these patients.

This study found rosiglitazone to be beneficial for the treatment of major depression. This result aligns with the findings of an earlier open‐label study in 2010 on 12 nondiabetic insulin‐resistant patients with depression. Participants receiving 8 mg/d of rosiglitazone add‐on to their usual treatments showed a significant decline in depression severity as measured by HDRS at weeks 6 and 12 compared to baseline, although there was a small sample size and open‐label noncontrolled design [11]. Moreover, previous preclinical studies suggested that rosiglitazone may have antidepressant effects. A 2023 study by Alhaddad et al. [27] indicated that administration of 10 or 30 mg/kg of rosiglitazone for 21 days reduced depressive behaviors in dexamethasone‐induced depressed mice, as measured by the forced swimming test (FST) and tail suspension test (TST). Two other studies on stress‐induced mice demonstrated that 5 mg/kg of rosiglitazone reversed depressive behaviors, as evidenced by FST and the open field test [28, 29]. In agreement with these results, Stupp et al. [30] revealed that administering 5 mg/kg of rosiglitazone to mice with lipopolysaccharide‐induced depressive‐like behavior resulted in an antidepressant effect in both the TST and FST. Furthermore, Sharma et al. [31] reported that daily application of 20 mg/kg of rosiglitazone for 5 weeks reduced immobility time in FST and exhibited antidepressant effects in diabetic mice. These findings are consistent with the results of Cheng et al. [10] and Ahmed et al. [32] regarding the effects of rosiglitazone in animal models of depression.

The antidepressant effects of other PPAR‐γ agonists have also been investigated. Two randomized, double‐blind clinical trials compared the antidepressant effects of 30 mg/d pioglitazone with placebo and another insulin‐sensitizer, metformin, in 40 MDD patients with or without metabolic comorbidities. Pioglitazone was superior to both groups in reducing the HDRS scores from baseline [14, 15]. Additionally, in an open‐label study, 23 patients with MDD were administered pioglitazone at doses ranging from 15 to 45 mg/d for 12 weeks. A significant reduction in symptom severity was reported in both clinician‐evaluated and patients self‐reported measures of depression severity [13]. In this regard, recent preclinical studies have also suggested antidepressant‐like effects of pioglitazone and another PPAR‐γ agonist, NP031115, in animal models of depression [33–35].

The antidepressant effects of PPAR‐γ agonists, including rosiglitazone, are believed to be mediated through multiple mechanisms. Impaired autophagy plays an essential role in the pathogenesis of depression. Studies have revealed that rosiglitazone can restore autophagy via activating the LKB1‐AMPK pathway in a PPAR‐γ‐manner and upregulation of AMPK‐dependent ULK1, an essential enzyme for the initiation of autophagy [28]. Astrocytic apoptosis and dysfunction in the prefrontal cortex (PFC) are also reported to be associated with depression [36]. Rosiglitazone increases insulin‐like growth factor 1 and insulin‐like growth factor 1 receptor levels, activating the Akt/CREB pathway, protecting astrocytes from apoptosis, and promoting neuronal axon plasticity [28].

Evidence suggests the crucial role of the adipose PPAR‐γ‐ adiponectin axis in regulating stress responses and emotion‐related behaviors, especially in depression. Adipose‐derived adiponectin significantly affects emotional responses. Its production is stimulated by the PPAR‐γ transcription factor, which decreases under chronic stress. Rosiglitazone, a PPAR‐γ agonist, enhances adiponectin levels in the plasma, acting as an antidepressant and an anxiolytic agent [6].

The neuroprotective and anti‐inflammatory effects of rosiglitazone may also contribute to its antidepressant activity. Inflammation is a major factor in depression, with elevated levels of pro‐inflammatory cytokines like TNF‐α, IL‐1β, and IL‐6 contributing to neuroinflammation, neurotransmitter dysregulation, and neuronal dysfunction in the PFC [37, 38]. Rosiglitazone reduces the aforementioned inflammatory cytokines in both serum and cerebrospinal fluid, inhibits microglial activation, suppresses neuroinflammation, and restores anti‐inflammatory cytokine activity [27, 28, 30]. Moreover, oxidative stress can cause neuronal damage, inflammation, and neurotransmitter imbalances, all of which lead to depressive symptoms [39]. Rosiglitazone reduces oxidative stress by decreasing malondialdehyde levels and enhancing superoxide dismutase activity [12].

There is another mechanism that can be discussed. Brain‐derived neurotrophic factor (BDNF) is a crucial protein in different brain regions, including the hippocampus and PFC, that plays an important role in neurogenesis and synaptic plasticity [40]. The BDNF levels decrease in depression, causing neuronal atrophy [41]. Recent studies suggest that rosiglitazone may prevent the reduction of BDNF in the hippocampus in animal models of depression, thereby alleviating depressive symptoms [12, 28, 34].

The term “efficacy” or similar expressions are often used in studies when *P*s fall below 0.05; however, this does not necessarily confirm the clinical significance of differences between the medication and the placebo [42]. For a minimal improvement that is clinically noticeable by practitioners, a reduction of at least seven points on the HDRS. Smaller changes, despite statistical significance, are associated with limited effect sizes [42, 43]. In this study, the effect size for the primary outcome was moderate. The debate over small effect sizes persists even for approved treatments [42, 43], but adjunctive therapies may enhance effect sizes through synergistic mechanisms or alternative pathways [44].

In this study, the remission rate between the rosiglitazone group and the placebo group was not statistically significant, which could be attributed to the short duration of the trial; 6 weeks may be an insufficient time for rosiglitazone to reach its complete therapeutic effects on a chronic inflammatory condition like depression [28, 45].

The current study found rosiglitazone to be safe and well‐tolerated in patients with moderate to severe MDD, with no significant difference in side effects compared to placebo. Compared to pioglitazone, which has a higher risk of bladder cancer, rosiglitazone was considered a safer option among PPAR‐γ agonists [46]. Cardiovascular risks, weight gain, bone fractures, and fluid retention are the serious side effects of rosiglitazone in former studies [47–49]. In a previous clinical study, one of the patients experienced weight gain [11]; however, in our study, no different adverse effects were reported. This conflict may be due to the shorter period of treatment or lower doses prescribed.

Although the lack of a diabetes diagnosis was not an exclusion criterion in this study, none of the participants had diabetes. In this study, blood glucose levels were measured at baseline and at weeks 2 and 4, on which rosiglitazone did not have any significant effect. This aligns with available evidence suggesting that the glucose‐lowering effects of rosiglitazone are primarily observed in individuals with preexisting insulin resistance, a hallmark of type 2 diabetes [50]. While rosiglitazone acts as a PPAR‐γ agonist, increasing insulin sensitivity [6], this mechanism may not be sufficient to significantly alter glucose homeostasis in individuals with normal insulin sensitivity.

Although this research offers significant advantages, such as a high‐quality design and accurate adjustment of baseline clinical characteristics, certain limitations should be acknowledged. First, the sample size of the study was relatively small. Second, the study duration was short. Third, due to ethical considerations, assessing the effects of monotherapy with rosiglitazone on depression was not done. Fourth, the patients’ follow‐up data recording after the treatment period was not done.

## 5. Conclusion

Rosiglitazone as an adjunct to sertraline was beneficial, safe, and well‐tolerated in patients with moderate to severe MDD. Larger‐scale, longer‐term studies should be conducted to validate these outcomes and examine the broader implications of rosiglitazone use in diverse populations. Future investigations should focus on monitoring glucose levels and inflammatory markers as well as considering the metabolic state of patients.

## Author Contributions

**Shahin Akhondzadeh and Mohammad-Reza Khodaei Ardakani**: conceptualization, project administration, supervision, funding acquisition, methodology, software. **Ahmad Shamabadi**, **Kimia Farahmand**, **Mohamadjavad Ershadmanesh**, and **Rozhin Moosavi**: methodology, writing – original draft, formal analysis, visualization, editing. **Reihane Karami**, **Sherinaz Sadati**, and **Fatemeh Rahiminejad**: data curation and investigation.

## Funding

This research was funded by a grant from the Tehran University of Medical Sciences (TUMS) to Professor Shahin Akhondzadeh (Grant 65858). TUMS did not participate in the study design, execution, data collection, analysis, interpretation, manuscript drafting, review, final approval, or decision to publish the findings.

## Disclosure

All authors read and approved the final manuscript.

## Ethics Statement

The study protocol was approved by the institutional research ethics committee (identifier IR.TUMS.MEDICINE.REC.1402.150).

## Consent

Written informed consent was obtained from all patients. They were informed of their right to withdraw consent at any time without impacting standard medical support or their professional relationship with clinicians.

## Conflicts of Interest

The authors declare no conflicts of interest.

## Data Availability

The data that support the findings of this study are available from the corresponding author upon reasonable request.

## Appendix A

**Table A1:** CONSORT 2010 checklist of information in reporting the trial^a^.

Section/Topic	Item number	Checklist item	Reported on section
Title and abstract			
	1a	Identification as a randomized trial in the title	Title
	1b	Structured summary of trial design, methods, results, and conclusions (for specific guidance see CONSORT for abstracts)	Abstract
Introduction			
Background and objectives	2a	Scientific background and explanation of rationale	1, paragraphs 1 to 4
	2b	Specific objectives or hypotheses	1, paragraph 4
Methods			
Trial design	3a	Description of trial design (such as parallel, factorial) including allocation ratio	2.1 and 2.6
	3b	Important changes to methods after trial commencement (such as eligibility criteria), with reasons	Not applicable
Participants	4a	Eligibility criteria for participants	2.3
	4b	Settings and locations where the data were collected	2.1
Interventions	5	The interventions for each group with sufficient details to allow replication, including how and when they were actually administered	2.4
Outcomes	6a	Completely defined prespecified primary and secondary outcome measures, including how and when they were assessed	2.7 and 2.8
	6b	Any changes to trial outcomes after the trial commenced, with reasons	Not applicable
Sample size	7a	How sample size was determined	2.5
	7b	When applicable, explanation of any interim analyses and stopping guidelines	Not applicable
Randomization:			
Sequence generation	8a	Method used to generate the random allocation sequence	2.6
	8b	Type of randomization; details of any restriction (such as blocking and block size)	2.6
Allocation concealment mechanism	9	Mechanism used to implement the random allocation sequence (such as sequentially numbered containers), describing any steps taken to conceal the sequence until interventions were assigned	2.6
Implementation	10	Who generated the random allocation sequence, who enrolled participants, and who assigned participants to interventions	2.6
Blinding	11a	If done, who was blinded after assignment to interventions (for example, participants, care providers, those assessing outcomes) and how	2.6
	11b	If relevant, description of the similarity of interventions	2.6
Statistical methods	12a	Statistical methods used to compare groups for primary and secondary outcomes	2.9
	12b	Methods for additional analyses, such as subgroup analyses and adjusted analyses	2.8 and 2.9
Results			
Participant flow (a diagram is strongly recommended)	13a	For each group, the numbers of participants who were randomly assigned, received intended treatment, and were analyzed for the primary outcome	Figure 1
	13b	For each group, losses, and exclusions after randomization, together with reasons	Figure 1
Recruitment	14a	Dates defining the periods of recruitment and follow‐up	2.1
	14b	Why the trial ended or was stopped	Not applicable
Baseline data	15	A table showing baseline demographic and clinical characteristics for each group	Table 1
Numbers analyzed	16	For each group, number of participants (denominator) included in each analysis and whether the analysis was by original assigned groups	Figure 1
Outcomes and estimation	17a	For each primary and secondary outcome, results for each group, and the estimated effect size and its precision (such as 95% confidence interval)	3.1 to 3.3 and Tables 2 to 4
	17b	For binary outcomes, presentation of both absolute and relative effect sizes is recommended	Not done
Ancillary analyses	18	Results of any other analyses performed, including subgroup analyses and adjusted analyses, distinguishing prespecified from exploratory	3.1.2
Harms	19	All important harms or unintended effects in each group (for specific guidance see CONSORT for harms)	3.3
Discussion			
Limitations	20	Trial limitations, addressing sources of potential bias, imprecision, and if relevant, multiplicity of analyses	4
Generalisability	21	Generalisability (external validity, applicability) of the trial findings	4
Interpretation	22	Interpretation consistent with results, balancing benefits and harms, and considering other relevant evidence	4
Other information			
Registration	23	Registration number and name of trial registry	2
Protocol	24	Where the full trial protocol can be accessed, if available	2
Funding	25	Sources of funding and other support (such as supply of drugs), role of funders	Funding

*Note:* Citation: Schulz KF, Altman DG, Moher D, for the CONSORT Group. CONSORT 2010 Statement: updated guidelines for reporting parallel‐group randomized trials. BMC Medicine. 2010;8:18. 2010 Schulz et al. This is an Open Access article distributed under the terms of the Creative Commons Attribution License (http://creativecommons.org/licenses/by/2.0), which permits unrestricted use, distribution, and reproduction in any medium, provided the original work is properly cited.

^a^We strongly recommend reading this statement in conjunction with the CONSORT 2010 Explanation and Elaboration for important clarifications on all items. If relevant, we also recommend reading CONSORT extensions for cluster ‐randomized trials, noninferiority and equivalence trials, nonpharmacological treatments, herbal interventions, and pragmatic trials. Additional extensions are forthcoming: for those and for up‐to‐date references relevant to this checklist, see www.consort-statement.org.
